# The Protective Effects of Water Extracts of Compound Turmeric Recipe on Acute Alcoholism: An Experimental Research Using a Mouse Model

**DOI:** 10.1155/2021/6641919

**Published:** 2021-01-13

**Authors:** Xian-ting Liang, Yan-yan Wang, Xiao-yu Hu, Shao-bo Wang

**Affiliations:** ^1^Department of Clinical Medicine, Chengdu University of Traditional Chinese Medicine, Chengdu, Sichuan 610075, China; ^2^Department of Infectious Diseases, Hospital of Chengdu University of Traditional Chinese Medicine, Chengdu, Sichuan 610072, China

## Abstract

Acute alcoholism (AAI) is a common emergency. Currently, there is a lack of preventive and therapeutic drugs with superior safety and efficacy. *Curcuma longa*, *Panax ginseng*, *Pueraria lobata*, *Pueraria* flower, and *Hovenia dulcis* Thunb., which are the components of compound turmeric recipe (CTR), are, respectively, used in China as adjuvant therapeutic agents for AAI and alcoholic liver injury, respectively. The purpose of this research was to investigate the effect of traditional compound turmeric recipe in anti-inebriation treatment and to identify its underlying mechanisms. The mice were administered with CTR mixture, and ethanol was subsequently given to mice by gavage. The effects of CTR on the righting reflex, 24-hour survival, drunken behavior, blood ethanol concentration, and pathological changes of liver are depicted. The activities of serum alanine aminotransferase (ALT), aspartate aminotransferase (AST), and alkaline phosphatase (ALP) were detected. Besides, the activities of tumor necrosis factor-*α* (TNF-*α*), interleukin-8 (IL-8), alcohol dehydrogenase (ADH), aldehyde dehydrogenase (ALDH), cytochrome P450 (P450), superoxide dismutase (SOD), and malondialdehyde (MDA) in the liver and the levels of *β*-endorphin (*β*-EP) and leucine enkephalin (LENK) in the brain were also measured. Our results demonstrated that CTR can increase the activities of ADH, ALDH, P450, and SOD and decrease the contents of TNF-*α*, IL-8, and MDA in the liver. In addition, it can decrease the activities of ALT, AST, and ALP in serum and *β*-EP and LENK activities in the brain. CTR showed effects on prevention of acute alcoholism, promoting wakefulness, and alleviating alcoholic liver injury, which were likely mediated by the above mechanisms.

## 1. Introduction

In recent years, alcoholism has become a major social problem in public health. More than 80% of heavy drinkers have a fatty liver to a certain extent, of which 10%–35% can develop into alcoholic hepatitis (AH), and 10%–20% of them will develop liver cirrhosis (AC) [1]. The latest WHO statistics show that drinking to excess kills 2.5 million people a year, which is one of the common high-risk factors of four major types of noncommunicable diseases. Alcohol was associated with 6.2% of male deaths and 1.1% of female deaths [2]. One-time heavy drinking can cause acute alcoholism. According to statistics, cardio-cerebrovascular disease, acute pancreatitis, and acute alcoholism are the three diseases with the highest incidence in holidays [3]. Acute alcoholism may cause central nervous system depression and metabolic abnormality, and severe patients may suffer from respiratory and circulatory failure and even death [4, 5]. On the one hand, how to avoid and prevent alcohol damage when drinking is a prominent research question, but currently there is no effective and safe drug to prevent AAI. On the other hand, first aid measures generally taken for severely poisoned patients include prohibition of alcohol, reducing central depression, promoting the excretion of alcohol in the digestive tract, and dissolving and removing alcohol metabolites. Current treatment methods mainly focus on blocking the inhibitory effect of excessive ethanol on the central nervous system and accelerating the metabolism of alcohol, but they have no direct effect on the pathogenesis of acute alcoholism. Based on the above two points, it is particularly important to find safe drugs to prevent and treat acute alcoholism. In China, the application of traditional Chinese medicine has a long history in the prevention and treatment of alcoholism and anti-inebriation [6]. In addition, natural drugs present wide pharmacological spectra on various targets and facilitate the recovery of physical conditions without toxic effects. The compound turmeric recipe used in this study comes from the hangover recipe of Professor Xiao-yu Hu. The prescription includes *Curcuma longa*, *Panax ginseng*, *Pueraria lobata*, *Pueraria* flower, and *Hovenia dulcis* Thunb. Regarding these Chinese medicines included in the prescription, Curcumin, the main active component of *Curcuma longa*, has various pharmacological effects such as antitumor, lipid-lowering, and antimicrobial effects, especially in antioxidation, scavenging free radicals, anti-inflammation, and protecting the liver [7]. Curcumin can inhibit the production of reactive oxygen species (ROS) and scavenge oxygen free radicals produced in the process of oxidation [8]; Curcumin alone can also improve the antioxidant activity and reduce liver damage in acute alcoholism mice [9]. Ginsenoside, the main active component of *Panax ginseng*, has anti-inflammatory and antioxidant effects [10]. Ginseng extract reduces the concentration of alcohol in rat plasma [11] and increases the blood alcohol clearance rate of humans [12]. *Panax ginseng* has a protective effect on acute alcoholism mice [13]. The extract of *Pueraria lobata* can reduce the harm of ethanol and its metabolites by promoting lipid metabolism and increasing the activities of antioxidant enzymes in the liver [14–16], inhibit the increase of intestinal permeability induced by ethanol, and improve the intestinal barrier dysfunction caused by ethanol [17]. The extract of *Pueraria* flower can increase the elimination rate constant of blood acetaldehyde and promote the elimination of acetaldehyde, but has no effect on blood ethanol concentration [18, 19]. Intragastric administration of Puerarin or intraperitoneal injection of its metabolites can reduce the blood alcohol concentration and mortality of ethanol poisoned rats [18]. *Hovenia dulcis* Thunb. has a preventive effect on alcoholic liver injury caused by excessive drinking in mice [20, 21]. The compound turmeric recipe combines the above Chinese herb medicines playing an active role in this study. RU-21 produced by American Psychiatrics Co., Ltd., is effective against drunkenness, and all its ingredients have been confirmed as safe by the US Food and Drug Administration. The mechanism of detoxification is as follows: its active ingredients can effectively reduce the oxidation of ethanol into acetaldehyde, accelerate the decomposition of acetaldehyde, and quickly and effectively reduce the content of acetaldehyde in the blood to detoxify. In this experiment, RU-21, a drug with sobering effect that is recognized to be safe and effective and to have nontoxic side effects, was selected as the positive drug to observe the sobering effect of Compound Turmeric Recipe.

The purpose of this research was to investigate the effect of the water extracts of compound turmeric recipe (CTR) on AAI mice and to identify its underlying mechanisms.

## 2. Materials and Methods

### 2.1. Animals

One hundred and twenty male Kunming (SPF) mice weighing 18 ± 2 g were bought from Dasuo Experimental Animal Study Center (Chengdu, China). The animals after quarantine were kept in the mouse feeding room of Internal Medicine Experimental Center of Chengdu University of Traditional Chinese Medicine. The room humidity is 40–70%, and they were kept at an environmental temperature (24–28°) under the diurnal conditions (light–dark: 06 : 00–20 : 00). Free eating and drinking were provided for mice. The mice were raised according to the Guiding Principles for the Care and Use of Animals. The Animal Care and Scientific Committee of Chengdu University of TCM approved all the animals' procedures.

### 2.2. Drugs

The formula for the compound turmeric recipe (CTR, one dose) is shown in Table 1. All the Chinese herbs in this recipe were produced by Sichuan Herbal Medicine Co., Ltd. (Sichuan, China). The herbs were identified as genuine medicinal materials by Professor Zhu-yun Yan from the Department of Authenticity Appraisal of Chengdu University of Traditional Chinese Medicine. These Chinese herbs were decocted, filtered, and condensed to 3.5 g·mL. The administration dose of 1 kg/mice = Mg/60 kg × 12.33 (M means a dose of traditional Chinese medicine, and 60 kg is the standard weight of an adult), and the gastric lavage dose of Chinese medicine in mice was 14 mL/kg. an RU-21 adult clinical dose (1 g *x* 6 = 6 g/d, equivalent dose in mice: 6 g/60 kg X 12.33 = 1.23 g/kg/d) was prepared. Before use, it was dissolved in normal saline, and the gastric lavage dose for mice was 14 mL/kg.

### 2.3. Materials and Chemicals

RU-21 (lot: 108671) was produced by American Psychiatrics Co., Ltd. Detection kits for ALDH (lot: 20120214), ADH (lot: 20120213), P450 (lot: 20120202), *β*-EP (lot: 20120202), LENK (lot: 20120220), SOD (lot: 20120216), MDA (lot: 20120212), TNF-*α* (lot: 120202), and IL-8 (lot: 120202) were provided by Nanjing Jiancheng Bioengineering Institute (Nanjing, China). Detection kits for ALT (lot: 120202), AST (lot: 120273), and ALP (lot: 120166) were obtained from Biosino Bio-technology Co., Ltd. (Beijing, China).

500 ml formaldehyde solution (lot: 111008) was provided by Chengdu Jinshan Chemical Reagent Co., Ltd. (Chengdu, China). 56° Liquor (lot: 20110923) was purchased from Beijing Hongxing Co., Ltd. (Beijing, China). All the Chinese herbs in this recipe were produced by Sichuan Lotus Decoction pieces Co., Ltd. (Sichuan, China). The herbs were identified as genuine medicinal materials by Professor Zhu-yun Yan from the Department of Authenticity Appraisal of Chengdu University of Traditional Chinese Medicine, according to the Pharmacopoeia of the People's Republic of China (2010).

### 2.4. Methods

#### 2.4.1. Observation of the Effects of CTR on Alcohol Tolerance Time, Time for Soberness, 24 h Survival Rate of Mice, and Drunkenness

The 120 SPF Kunming mice were fed for one week in the mouse feeding room of Chengdu University of Traditional Chinese Medicine. Then, the mice were randomized into control group, model group, RU-21 group, and CTR group (compound turmeric recipe group), with 30 mice in each group. Before the experiment, the mice were fasted for 12 hours, but they were allowed to drink freely. The RU-21 group and CTR group were administered with RU-21 (12.33 g/kg) and water extracts of compound turmeric recipe (55.48 g/kg), respectively. The control group and model group were given the same volume of normal saline. Half an hour later, except for the control group, all groups were administered with 14 ml/kg ethanol in order to establish the mice models of acute alcoholism, and the control group was replaced by the isometric normal saline. Then, we observed the following items: alcohol tolerance time, time for soberness, 24 h survival rate of mice, and drunkenness.

*(1) Alcohol Tolerance Time*. The time of gavage and that of drunkenness were recorded for the purpose of calculating the tolerance time. The sign of judging the drunkenness of mouse was the disappearance of righting reflex.

*(2) The Time for Soberness (Sober-Up Time)*. The time of drunkenness and that of sobering up were recorded for the purpose of calculating the time for soberness, the duration from the start of the disappearance of righting reflex to the reappearance of righting reflex. The sign of judging the drunkenness of mouse was the disappearance of righting reflex. After the mice were drunk, we let the mice lie on their backs in the cage. If the mice could keep lying on their backs for over 30 seconds, this was considered as the disappearance of righting reflex; otherwise, they were not drunk [11, 22].

#### 2.4.2. Determination of the Concentration of Ethanol in the Blood of Mice; the Levels of ADH, ALDH, and P450 in Liver Tissue; and the Concentrations of *β*-EP and LENK in Brain Tissue

According to each time period, another 288 male SPF Kunming mice were randomly divided into 6 groups, with 48 mice in each group. In addition, each group was further divided into control group, model group, RU-21 group, and CTR group, with 12 mice in each group. The specific intervention methods were the same as before. After ethanol intake, the blood samples were collected from iliac veins at 0.5 h, 1 h, 1.5 h, 2 h, 3 h, and 6 h. The samples were placed in sodium heparin tubes, and they were stored in a −20°C refrigerator for testing.

After 6 hours of intragastric administration of ethanol or normal saline, the blood samples were collected from the iliac vein and centrifuged at 3000 r/min for 5 minutes, and the serum was collected. Oxidized forms of alcohol dehydrogenase (ADH)-nicotinamide adenine dinucleotide (NAD) were put into the blood standard solutions of different ethanol concentrations. The absorbance of the above reaction mixture was documented at 340 nm, and the standard curve of ethanol concentration was drawn. The regression equation of ethanol concentrations was presented as *C* = 1237.6A − 11.862 (*r* = 0.967) through the above standard curve. The formula was used to calculate the concentration of ethanol in the blood of mice.

After 6 hours of intragastric administration of ethanol or normal saline, the abdominal skin was cut along the midline of the abdomen, and liver tissue was taken. The left lobe of the liver was washed repeatedly with normal saline and then dried with filter paper. Around 0.3 g liver tissue was cut off and used to prepare liver homogenate by 1 : 9 addition of normal saline, and the 10% liver homogenate was prepared by glass homogenizer. The 10% liver homogenate was centrifuged at 3000 r/min for 15 min at low temperature, and the supernatant was collected for detection. The levels of ADH and ALDH in liver homogenate were determined by ELISA, in accordance with the kit instruction manual. The specific operation methods of the ELISA include standard dilution, sample addition, incubation, liquid mixing, washing, enzyme addition, incubation, washing, coloration, reaction termination, and determination of the absorbance. The determination should be carried out within 15 minutes after the addition of termination fluid. The level of P450 in liver tissue homogenate was also detected by ELISA, and the preparation procedures of the liver tissue homogenate and ELISA method are the same as before. The preparation procedures of 10% brain tissue homogenate were the same as the liver tissue homogenate, and the brain homogenate was put into a −70°C refrigerator for determination. The concentrations of *β*-EP and LENK in brain homogenate were also detected by ELISA, and the specific methods were the same as above.

#### 2.4.3. Determination of Serum ALT, AST, and ALP Concentrations and Liver Tissue TNF-*α*, IL-8, SOD, and MDA Concentrations in Mice and Observation of the Histopathological Features of Liver under an Optical Microscope

The preparation procedures of the liver tissue homogenate and experimental methods were the same as above. After 6 hours of intragastric administration of ethanol or normal saline, the blood samples were collected from the iliac vein and centrifuged at 3000 r/min for 5 minutes to get serum. The activities of ALT, AST, and ALP in serum were detected by automatic biochemical analyzer according to the instructions of detection kits. The preparation methods of 10% liver tissue homogenate were consistent with the previous experiment. The 10% liver tissue homogenate was centrifuged at 3000 r/min for 15 min, and the supernatant was stored at low temperature for determination of SOD and MDA concentrations. According to Buege [23], SOD activity was determined by xanthine oxidase, and MDA content in liver was determined by therapeutic acid-reactive substances (T-BARS) methods. The preparation methods of the specimen and experimental procedures were the same as before. Besides, the levels of TNF-*α* and IL-8 in liver tissue homogenate were detected by ELISA. The liver tissue was stained through hematoxylin and eosin (HE): the same tissue in the right lobe of liver was fixed in 10% formaldehyde immediately and embedded with paraffin. The paraffin block was cut into 5 *μ*m thick slices by a microtome and stained by hematoxylin and eosin (HE). The histopathological features of the liver were observed under an optical microscope. Three researchers were blinded to allocation arm of the mice, and they read all the slides. The researchers used the alcoholic liver disease grading and staging criteria [24] to classify fat variables and inflammation in liver, with grade 1 indicating no identifiable damage and grade 4 indicating severe damage.

### 2.5. Statistical Analysis

Statistical software SPSS17.0 was used for statistical analyses. The measurement data was expressed as mean ± SEM. The enumeration data was compared by chi-square test.

Analysis of variance was used to analyze the differences among groups. The log-rank test was applied to compare the survival rate, and the Kaplan–Meier method was utilized for survival analysis. All statistical tests were two-sided. If *p* < 0.05, the differences among data were considered to have statistical significance.

## 3. Results

### 3.1. Effect of CTR on Alcohol Tolerance Time

**Figure 1:**
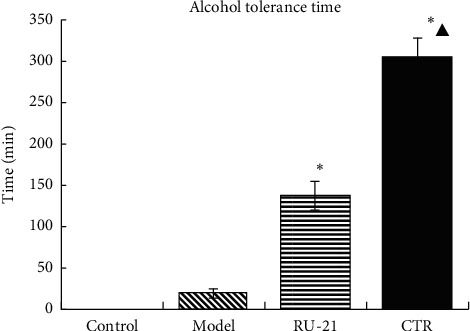
Comparison of the alcohol tolerance time among groups. ^*∗*^*p* < 0.01 compared with the model group, ^▲^*p* < 0.01 compared with the RU-21 group.

Compared with the model group, the CTR group and RU-21 group had significantly longer alcohol tolerance time. In addition, there was a significant difference in alcohol tolerance time between the CTR group and the RU-21 group, indicating that the effect of the CTR was stronger (Table 2, Figure 1). The results showed that CTR can prolong the tolerance time.

### 3.2. Effect of CTR on Sober-Up Time

**Figure 2:**
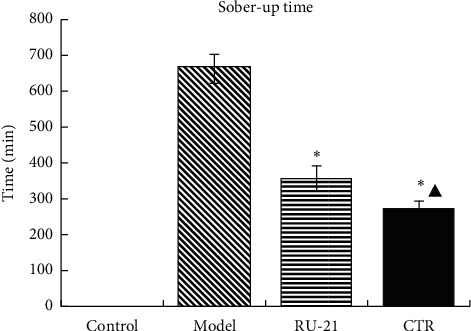
Comparison of the sober-up time among groups. ^*∗*^*p* < 0.01 compared with the model group, ^▲^*p* < 0.01 compared with the RU-21 group.

Compared with the model group, the CTR group and the RU-21 group had significantly shorter sober-up time. In addition, compared with RU-21 group, the CTR could significantly shorten the sober-up time (Table 3 and Figure 2). The results showed that CTR can significantly shorten the sober-up time.

### 3.3. Effect of CTR on 24-Hour Survival

**Figure 3:**
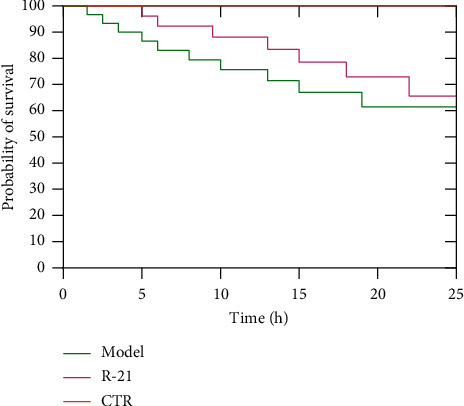
The mice survival curves chart in 24 hours. Model: the group cured with alcohol only. RU-21: the positive control group cured with RU-21 before alcohol. CTR: the experiment group cured with compound turmeric recipe before alcohol.

The 24-hour survival rate is presented in Figure 3. It was found that the 24 h survival rate of mice in the CTR group was significantly higher than that of the model group (*X*^2^ = 9.596, *p* < 0.01). The results showed that CTR can improve the 24-hour survival rate, and the efficacy of CTR was better than RU-21.

### 3.4. Effect of CTR on Drunken Behavior in Mice

**Figure 4:**
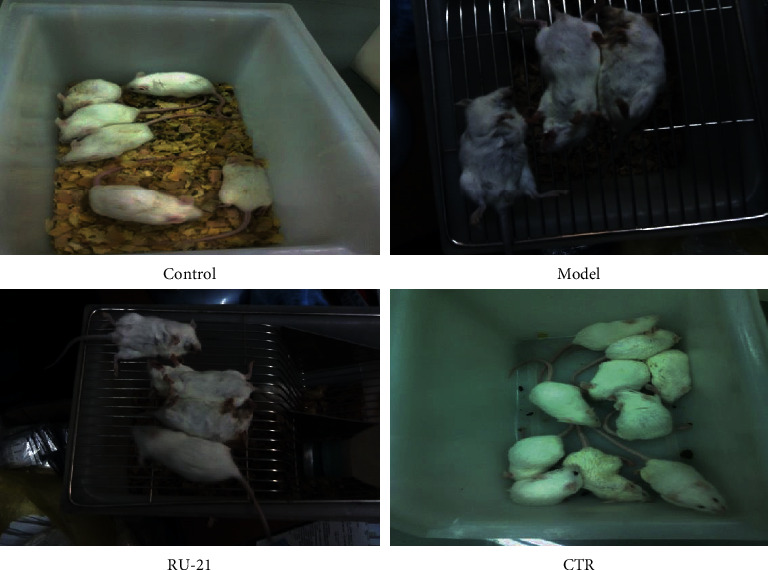
The drunken behavior of mice after 1 hour of intragastric administration of ethanol.

The mice in control group had agile activities, positive righting reflex, and normal bowel movements. After taking alcohol, the mice in model group successively showed excitatory hyperactivity, slow breathing and heart rate, disappearance of righting reflex, and fecal incontinence. After prophylactic administration, the mice in CTR group developed the above symptoms significantly later than the model group and RU-21 group. The mice in CTR group can resume drinking and eating after a period of time and gradually become normal (Figure 4).

### 3.5. Effect of CTR on Blood Ethanol Concentration

**Figure 5:**
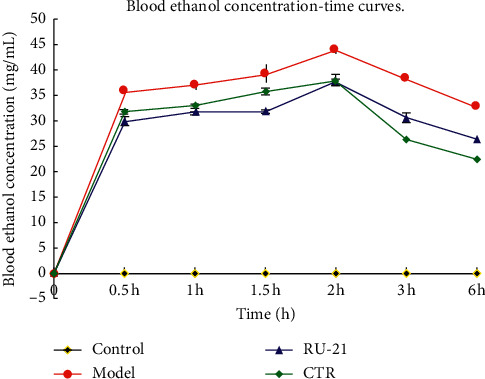
The mice blood ethanol concentration-time curves.

The blood ethanol concentration of mice in CTR group and RU-21 group was lower than that in model group at 0.5 h, 1 h, 1.5 h, 2 h, 3 h, and 6 h after alcohol intake; *p* < 0.01. Moreover, compared with the RU-21 group, the CTR group showed significantly reduced blood ethanol concentrations at 2 h, 3 h, and 6 h after alcohol intake; *p* < 0.01 (Table 4, Figure 5). The results showed that CTR can more significantly reduce the blood ethanol concentration of mice than RU-21.

### 3.6. Effect of CTR on ADH, ALDH, and P450 Activities in Liver Homogenate

**Figure 6:**
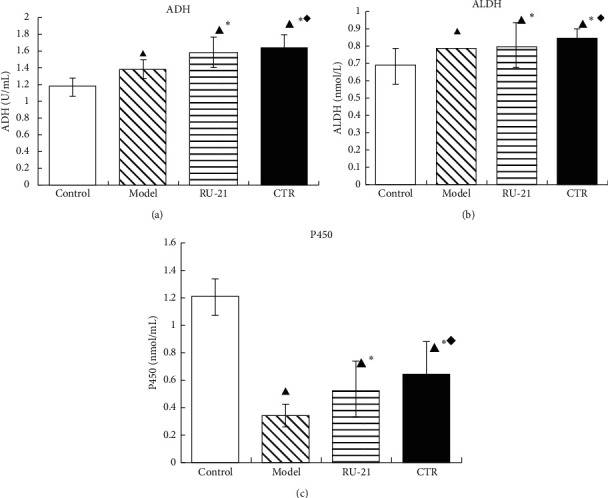
Comparison of acute alcoholism in mice: ADH, ALDH, and P450 levels among groups. (a) ADH; (b) ALDH; (c) P450 control: the group without alcohol or any drugs. Model: the group cured with alcohol only. RU-21: the positive control group cured with RU-21 before alcohol. CTR: the experiment group cured with the CTR before alcohol. ^▲^*p* < 0.05 compared with the control group, ^*∗*^*p* < 0.05 compared with the model group, and ^◆^*p* < 0.05 compared with the RU-21 group.

The activities of ADH, ALDH, and P450 in CTR group were significantly higher than those in model group and RU-21 group; *p* < 0.05 (Table 5, Figure 6). As shown in Table 4, the differences in ALDH activity were relatively small between the model group, RU-21 group, and CTR group, but the RU-21 group was still lower than the CTR group; *p* < 0.05. The results showed that preadministration of CTR enhanced the ADH, ALDH, and P450 activities in liver after alcohol intake.

### 3.7. Effect of CTR on *β*-EP and LENK Levels in Brain Tissue Homogenate

**Figure 7:**
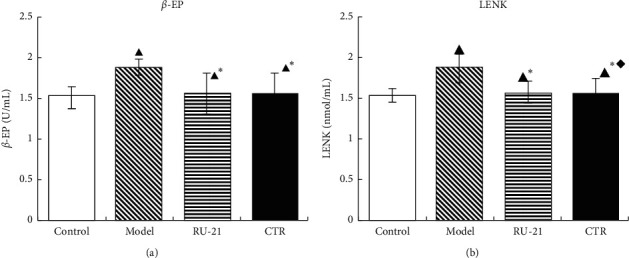
Comparison of acute alcoholism in mice: *β*-EP and LENK levels among groups. (a) *β*-EP; (b) LENK. Control: the group without alcohol or any drugs. Model: the group cured with alcohol only. RU-21: the positive control group cured with RU-21 before alcohol. CTR: the experiment group cured with the CTR before alcohol. ^▲^*P* < 0.05 *p* < 0.05 compared with the control group, ^*∗*^*p* < 0.05 compared with the model group, and ^◆^*p* < 0.05 compared with the RU-21 group.

The levels of *β*-EP and LENK in the brain were significantly increased after the administration of alcohol. Compared with the model group, the CTR group and RU-21 group had significantly lower levels of *β*-EP and LENK; *p* < 0.05 (Table 6, Figure 7). The results showed that CTR significantly reduced the brain *β*-EP and LENK levels in mice models of acute alcoholism.

### 3.8. Effect of CTR on ALT, AST, and ALP Activities in Serum of Mice

**Figure 8:**
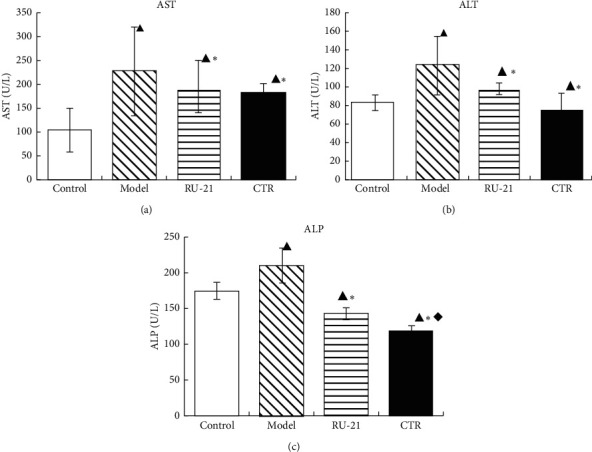
Comparison of acute alcoholism in mice: the changes of liver enzyme among groups. (a) ALT; (b) AST; (c) ALP. Control: the group without alcohol or any drugs. Model: the group cured with alcohol only. RU-21: the positive control group cured with RU-21 before alcohol. CTR: the experiment group cured with the CTR before alcohol. ^▲^*p* < 0.05 compared with the control group, ^*∗*^*p* < 0.01 compared with the model group, and ^◆^*p* < 0.05 compared with the RU-21 group.

As shown in Table 7 and Figure 8, the serum ALT, AST, and ALP activities in CTR group and RU-21 group were significantly lower than those in model group; all *p* < 0.05. Compared with the RU-21 group, the CTR group showed significantly decreased ALP activity; *p* < 0.05. However, there was no significant difference between the RU-21 group and the CTR group in AST. The results showed that CTR reduced the serum ALT, AST, and ALP activities in mice models of acute alcoholism.

### 3.9. Effect of CTR on SOD and MDA Activities in Liver Tissue Homogenate

**Figure 9:**
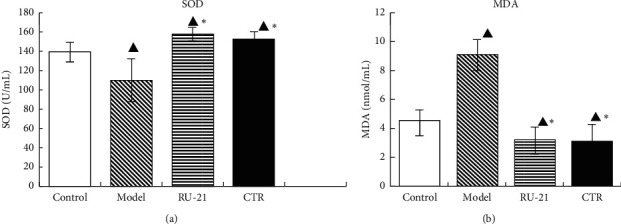
Comparison of acute alcoholism in mice: the liver SOD and MDA levels among groups. (a) SOD; (b) MDA. Control: the group without alcohol or any drugs. Model: the group cured with alcohol only. RU-21: the positive control group cured with RU-21 before alcohol. CTR: the experiment group cured with the CTR before alcohol. ^▲^*p* < 0.05 compared with the control group and ^*∗*^*p* < 0.05 compared with the model group.

The liver SOD and MDA activities are presented in Table 8 and Figure 9. From the results, the model group has lower SOD activity and higher MDA activity than the control group; *p* < 0.05. Compared with model group, CTR group had significantly higher liver SOD activity and lower MDA content; *p* < 0.05. Both RU-21 and CTR treatment could increase liver SOD activity and decrease liver MDA activity.

### 3.10. Effect of CTR on TNF-*α* and IL-8 Levels in Liver Tissue Homogenate

**Figure 10:**
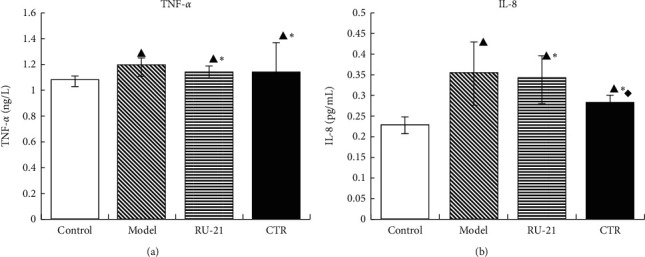
Comparison of acute alcoholism in mice: TNF-*α* and IL-8 levels among groups. (a) TNF-*α*; (b) IL-8. Control: the group without alcohol or any drugs. Model: the group cured with alcohol only. RU-21: the positive control group cured with RU-21 before alcohol. CTR: the experiment group cured with the CTR before alcohol. ^▲^*p* < 0.05 compared with the control group, ^*∗*^*p* < 0.05 compared with the model group, and ^◆^*p* < 0.05 compared with the RU-21 group.

As presented in Table 9 and Figure 10, the levels of TNF-*α* and IL-8 in model group were higher than those in control group; *p* < 0.05. Compared with model group, CTR group and RU-21 group had lower levels of TNF-*α* and IL-8; *p* < 0.05. There was no significant difference between CTR group and RU-21 group in TNF-*α*, but the decrease of IL-8 activity in CTR group was more significant than that in RU-21 group; *p* < 0.05. CTR significantly reduced the levels of TNF-*α* and IL-8 in liver tissue.

### 3.11. Histopathological Features of the Liver

#### 3.11.1. Observation under the Microscope

**Figure 11:**
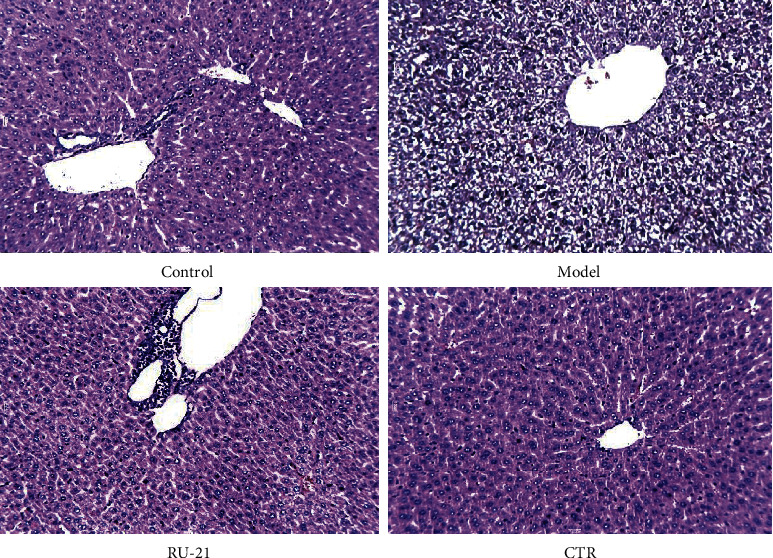
Histopathological features of the liver with H&E staining.

The mice in the control group had normal hepatocyte structure and orderly arrangement of liver cords. There was no obvious steatosis, necrosis, or inflammatory cell infiltration in hepatocytes. In the model group, the hepatic lobule structure was disordered, with obvious swelling around the central vein of hepatocytes. The cytoplasm is transparent with balloon-like changes, necrotic areas, and infiltration of inflammatory cells. Compared with control group, parts of hepatocytes in RU-21 group and CTR group were swollen. The boundary between hepatocytes was clear with light staining of cytoplasm. A small number of inflammatory cells were found in individual portal areas, which were fewer than those of the model group. Under the microscope magnifying of 200, 10 microscopic vision fields were randomly captured on each pathological slice, and the pathological slices were graded according to the relevant standards [24] (Figure 11). Rank sum test was used to compare the differences between the groups. CTR could significantly reduce the liver injury caused by alcohol (Figure 11: *X*^2^ = 11.719; *p* < 0.01).

## 4. Discussion

The results showed that the CTR treatment prolonged the alcohol tolerance time, significantly shortened the time for soberness, and prolonged the 24-hour survival rate of mice with acute alcoholism. In addition, the CTR treatment delayed the appearance of intoxication symptoms in mice with acute alcoholism and weakened the intoxication symptoms of mice such as hyperactivity. In the in vivo study, the CTR treatment increased the activities of ADH, ALDH, and P450 and decreased *β*-EP and LENK levels. Besides, it also reduced ALT, AST, and ALP activities, increased SOD activity, and decreased MDA, TNF-*α*, and IL-8 activities. Moreover, the ethanol concentration in blood was reduced after the CTR treatment, which could accelerate the metabolism of alcohol/aldehyde and/or inhibit the absorption of alcohol.

As a vital physical function, behavioral function is thought to be the most sensitive and complex function. At present, it is helpful to understand the mechanism of medicine actions to study behavioral reaction of animals, rather than the chemical mechanism of brain. Behavioral function is an important function of organism, which is regarded as the most complicated and sensitive function. Studying the behavioral response of animals, rather than the brain chemistry mechanism, is conducive to understanding the mechanism of drug action. The animal's sense of discomfort is manifested objectively and truly through behavior changes. The disappearance of righting reflex is regarded as an indicator of drunkenness [25]. The behavior experiment was conducted to study time for soberness and alcohol tolerance time, and the results showed that CTR had better effects on “promoting revival” and “preventing drunkenness.” The 24-hour survival rate was significantly improved after treatment with CTR. CTR is better than RU-21 in preventing drunkenness, promoting sobering up, and improving 24-hour survival rate.

In humans and mammals, alcohol dehydrogenase oxidation system, which includes alcohol dehydrogenase (ADH) and aldehyde dehydrogenase (ALDH), and micro ethanol oxidation system (MEOS) are two main pathways for liver metabolism of ethanol. About 8%-10% of ethanol is metabolized into acetaldehyde by microsomal ethanol oxidase (EO) and about 90% by ADH [26]. Then, acetaldehyde is metabolized into acetic acid under the action of ALDH and furthermore into CO_2_ and H_2_O through the TCA (tricarboxylic acid) cycle [27, 28]. Accordingly, ALDH and ADH are the two most important enzymes in the whole process of ethanol metabolism in the liver. Relevant study shows that acute or chronic ethanol consumption can significantly inhibit the activities of ALDH and ADH [29]. The microsomal ethanol oxidation system (MEOS) in hepatocytes requires the participation of cytochrome P450 (CYP450) and nicotinamide adenine dinucleotide phosphoric acid (NADPH) to metabolize ethanol to acetaldehyde. Alcoholism increases the amount of acetaldehyde and consumes more liver P450 enzymes, resulting in insufficient supply of P450 enzymes in the body, and the liver's ability to metabolize alcohol is reduced, resulting in the damage of liver cells by alcohol [30, 31]. Therefore, the metabolism of MEOS can be reflected indirectly by observing the activity of P450. This study showed that ADH, ALDH, and P450 activities were significantly inhibited in acute alcoholism model mice. The CTR can increase the activities of ADH and ALDH, accelerate the oxidative metabolism of ethanol and acetaldehyde in vivo, and reduce the toxicity of acetaldehyde; moreover, it can significantly increase the P450 activity, activate the MEOS, and accelerate the metabolism of ethanol. In addition, CTR has a better effect on alcohol metabolism than RU-21. The ethanol concentration curve after alcohol intake also confirmed the above viewpoints.

Alcohol intoxication can cause the anterior pituitary to release a large amount of *β*-EP. A high level of *β*-EP can directly inhibit the central nervous system. At the same time, *β*-EP can cause cerebral ischemic injury by inhibiting prostaglandin production and adenylate cyclase activity [32]. High level of *β*-EP also inhibits ATP metabolism, promotes the production of oxygen free radicals, and damages brain cells [33]. LENK is an opioid peptide. When the body is stimulated by acute alcohol, the synthesis, storage, and secretion of related opioid peptides will change significantly, which may even make the opioid peptide become a self-damaging factor in the body and participate in the pathological process of body injury or disease [34]. The results showed that CTR can significantly inhabit the release of LENK and *β*-EP, alleviate central nervous system inhibition, and exert a good antialcohol effect. In addition, CTR inhibits the release of *β*-EP and LENK more significantly than RU-21.

When hepatocytes are damaged, the permeability of hepatocyte membrane increases, ALT, AST, and ALP can enter blood in large quantities, and their activities are increased. Therefore, high transaminase generally indicates liver damage [35]. The study showed that CTR can significantly reduce the serum ALT, AST, ALP activities. The study indicated that it has a protective effect on alcohol-induced acute liver injury in mice. The mechanism was likely related to stabilizing the hepatic cellular membrane against alcohol-induced liver injury.

Alcohol in the body increases the level of reactive oxygen species, decreases levels of antioxidants of cells, and produces oxidative stress [36]. SOD is one of the most important antioxidant enzymes in vivo, which can protect against oxidative damage [37, 38]. Superoxide dismutase (SOD) can scavenge the body's excessive free radicals O2- to relieve the body damage caused by free radicals oxidizing certain components in the body [39, 40]. MDA is the final product of free radical acting on lipid, which can reflect the degree of lipid peroxidation in the human body and indirectly reflect the degree of cell damage [41]. During acute alcoholism, large amounts of oxygen free radicals are generated, MDA content increases, SOD consumption increases, and SOD activity decreases significantly. Brain tissue is the main organ of oxidative damage, which leads to abnormal neurobehavior after acute alcoholism. The results showed that CTR could significantly increase SOD activity and decrease MDA content. Besides, it could mediate the metabolism levels of oxygen free radicals in the liver, raise body antioxidation, and reduce oxidative stress, so as to protect the liver.

TNF-*α* and IL-8 are important inflammatory cytokines that mediate the body's inflammatory responses and have important roles in the physiological functions and pathological processes. Alcohol stimulates monocyte macrophages and increases the activation of TNF-*α* and IL-8, resulting in the imbalance of cytokine network. TNF-*α* cooperates with other cytokines to mediate inflammatory reaction and damage liver cells [42]. In recent years, animal experiments and clinical studies showed that the changes of inflammatory cytokines are closely related to the occurrence of alcoholic liver disease [43]. The results also showed that there were high levels of TNF-*α* and IL-8 in the liver tissues of mice with acute alcoholism, which mediated inflammatory reactions and damaged liver cells, and CTR significantly reduced the activities of TNF-*α* and IL-8, which suggested that CTR can effectively reduce the activities of TNF-*α* and IL-8 and decrease the levels of inflammatory mediators, so as to reduce the inflammatory injury of liver in acute alcoholism.

## 5. Conclusion

1. The results showed that compound turmeric recipe could promote wakefulness and prevent acute alcoholism: it could significantly prolong alcohol tolerance time of AAI mice, shorten the time for soberness, and improve the 24 h survival rate of mice. First of all, its mechanism was likely related to the reduction of blood alcohol concentration: namely, compound turmeric recipe induced the increase of the activities of ADH, ALDH, and P450 in the liver, thus accelerating the metabolism of alcohol. In addition, the recipe reduced the activities of *β*-EP and LENK in brain tissue, thus reducing the inhibitory effect of alcohol on the central nervous system.
2. Compound turmeric recipe has a protective effect on alcohol-induced liver injury: it could significantly reduce the activities of serum ALT, AST, and ALP of AAI mice. The mechanisms of liver protection were likely related to inhibiting the activities of TNF-*α*, IL-8, and MDA in liver, enhancing SOD content. That is to say, it is related to alleviating the liver free radical damage caused by alcohol, raising the antioxidant capability in the body and reducing the production of inflammatory mediators.

## Figures and Tables

**Table 1:** The ingredients of compound turmeric recipe (CTR, one dose).

Chinese medicines	Medicinal parts	Origin	Amount in preparation (g)
*Curcuma longa* L. (Jianghuang)	Radix	Sichuan province	40
*Panax ginseng* C. A. Meyer (Renshen)	Radix	Jilin province	50
*Pueraria lobata* (Willd.) Ohwi (Gegen)	Radix	Sichuan province	40
*Pueraria* flower (Gehua)	Flower	Sichuan province	40
*Hovenia dulcis* Thunb. (Zhijuzi)	Seed	Shaanxi province	30

**Table 2:** Comparison of the alcohol tolerance time and ebriety rate among groups ($\bar{x}$±S, *n* = 30).

Groups	*N*	Tolerance time ($\bar{x}$±S, min)	Ebriety numbers or rate (*n*, %)
Control	30	—	—
Model	30	19.23 ± 6.29	30 (100)
RU-21	30	137.1 ± 17.65^*∗*^	21 (70)
CTR	30	304.5 ± 23.72^*∗*^^▲^	10 (33)

^*∗*^ *p* < 0.01 compared with the model group; ^▲^*p* < 0.01 compared with the RU-21 group.

**Table 3:** Comparison of the sober-up time and rate of death among groups ($\bar{x}$ ± S, *n* = 30).

Groups	*n*	Sober-up time ($\bar{x}$±S, min)	Death numbers or rate (*n*, %)
Control	30	—	—
Model	20	661.43 ± 38.56	10 (33)
RU-21	23	351.9 ± 35.93^*∗*^	7 (23)
CTR	30	265.50 ± 27.2^*∗*^^▲^	0

^*∗*^ *p* < 0.01 compared with the model group; ^▲^*p* < 0.01 compared with the RU-21 group.

**Table 4:** Comparison of the blood ethanol concentration in mice with acute alcoholism (unit: mg·mL-1).

Groups hour	Control	Model	RU-21	CTR
0	0	0	0	0
0.5	0	35.93 ± 0.87	30.02 ± 0.28^*∗*^	31.47 ± 0.67^*∗*^^▲^
1	0	37.19 ± 0.44	31.84 ± 0.4^*∗*^	32.64 ± 0.6^*∗*^^▲^
1.5	0	39.02 ± 0.32	33.85 ± 0.52^*∗*^	35.77 ± 0.66^*∗*^^▲^
2	0	44.12 ± 1.64	38.95 ± 0.71^*∗*^	37.76 ± 1.11^*∗*^^▲^
3	0	38.23 ± 0.58	30.65 ± 0.42^*∗*^	27.52 ± 1.58^*∗*^^▲^
6	0	32.7 ± 0.62	26.31 ± 0.37^*∗*^	23.81 ± 1.03^*∗*^^▲^

^*∗*^ *p* < 0.01 compared with the model group; ^▲^*p* < 0.05 compared with RU-21 group.

**Table 5:** Comparison of acute alcoholism in mice: ADH, ALDH, and P450 levels among groups ($\bar{x}$±S, *n* = 12).

Groups	n	ADH (U/mL)	ALDH (nmol/mL)	P450 (nmol/mL)
Control	12	1.171 ± 0.104	0.6847 ± 0.1	1.2077 ± 0.13
Model	9	1.372 ± 0.119^▲^	0.7822 ± 0.06^▲^	0.3449 ± 0.08^▲^
RU-21	11	1.587 ± 0.186^▲^^*∗*^	0.8016 ± 0.13^▲^^*∗*^	0.5378 ± 0.2^▲^^*∗*^
QWJH	12	1.628 ± 0.174^▲^^*∗*^^◆^	0.8398 ± 0.06^▲^^*∗*^^◆^	0.6356 ± 0.25^▲^^*∗*^^◆^

^▲^ *p* < 0.05 compared with control group, ^*∗*^*p* < 0.05 compared with model group, and ^◆^*p* < 0.05 compared with RU-21 group.

**Table 6:** Comparison of acute alcoholism in mice: *β*-EP and LENK levels among groups ($\bar{x}$±S, *n* = 12).

Groups	*n*	*β*-EP (U/mL)	LENK (nmol/mL)
Control	12	1.509 ± 0.18	1.665 ± 0.08
Model	9	1.876 ± 0.09^▲^	1.865 ± 0.18^▲^
RU-21	11	1.551 ± 0.25^▲^^*∗*^	1.765 ± 0.13^▲^^*∗*^
CTR	12	1.549 ± 0.25^▲^^*∗*^	1.681 ± 0.21^▲^^*∗*^^◆^

^▲^ *p* < 0.05 compared with the control group, ^*∗*^*p* < 0.05 compared with the model group, and ^◆^*p* < 0.05 compared with the RU-21 group.

**Table 7:** Comparison of acute alcoholism in mice: the activities of ALT, AST, and ALP among groups ($\bar{x}$±S, *n* = 12).

Groups	*n*	ALT (U/L)	AST (U/L)	ALP (U/L)
Control	12	83.25 ± 8.61	103.7 ± 44.83	176.0 ± 12.3
Model	9	123 ± 31.35^▲^	325.5 ± 52.18^▲^	211.8 ± 24.54^▲^
RU-21	11	97.5 ± 5.55^▲^^*∗*^	193.5 ± 55.39^▲^^*∗*^	143.8 ± 9.3^▲^^*∗*^
CTR	12	75.25 ± 17.77^▲^^*∗*^	181 ± 20.52^▲^^*∗*^	121 ± 4.96^▲^^*∗*^^◆^

^▲^ *p* < 0.05 compared with the control group, ^*∗*^*p* < 0.01 compared with the model group, and ^◆^*p* < 0.05 compared with the RU-21 group.

**Table 8:** Comparison of acute alcoholism in mice: liver SOD and MDA levels among groups ($\bar{x}$±S, *n* = 12).

Groups	*n*	SOD (U/mL)	MDA (nmol/mL)
Control	12	139.09 ± 10.41	4.51 ± 0.81
Model	9	109.87 ± 22.01^▲^	9.13 ± 1.03^▲^
RU-21	11	157.46 ± 7.13^▲^^*∗*^	3.2 ± 0.98^▲^^*∗*^
CTR	12	151.97 ± 7.72^▲^^*∗*^	3.08 ± 1.3^▲^^*∗*^

^▲^ *p* < 0.05 compared with the control group and ^*∗*^*p* < 0.05 compared with the model group.

**Table 9:** Comparison of acute alcoholism in mice: TNF-*α* and IL-8 levels among groups ($\bar{x}$±S, *n* = 12).

Groups	*N*	TNF-*α* (ng/L)	IL-8 (pg/mL)
Control	12	1.067 ± 0.042	0.2279 ± 0.02
Model	9	1.18 ± 0.073^▲^	0.3562 ± 0.08^▲^
RU-21	11	1.137 ± 0.043^▲^^*∗*^	0.3428 ± 0.06^▲^^*∗*^
CTR	12	1.1354 ± 0.225^▲^^*∗*^	0.2836 ± 0.02^▲^^*∗*^^◆^

^▲^ *p* < 0.05 compared with the control group, ^*∗*^*p* < 0.05 compared with the model group, and ^◆^*p* < 0.05 compared with the RU-21 group.

## Data Availability

The datasets supporting the conclusions of the article are presented in the main paper. Plant materials used in this study have been identified in the Department of Authenticity Appraisal of Chengdu University of Traditional Chinese Medicine where voucher specimens are deposited.

## Supplementary Materials

Supplementary Materials The supplementary file includes the graphical abstract.
