# Supplementary material for: The Protective Effects of Water Extracts of Compound Turmeric Recipe on Acute Alcoholism: An Experimental Research Using a Mouse Model
Source: Evid Based Complement Alternat Med. 2021 Jan 13;2021:6641919. doi: 10.1155/2021/6641919 (PMC7817264; doi:10.1155/2021/6641919)
Supplement: Supplementary Materials — The supplementary file includes the graphical abstract. [file 6641919.f1.docx]

**GRAPHICAL ABSTRACT**


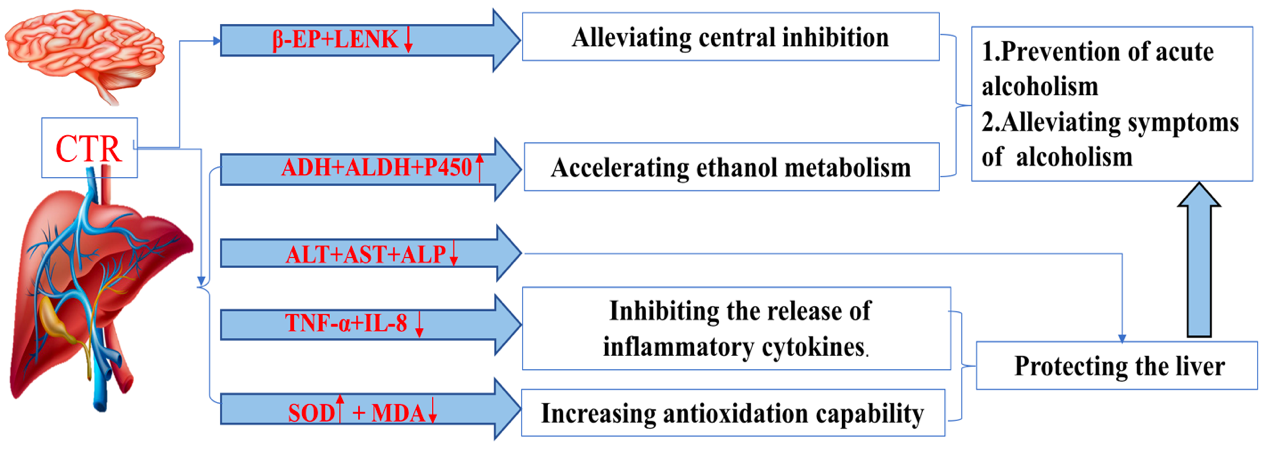


Compound Turmeric Recipe (CTR) could "prevent drunkenness", and "promote wakefulness ", and significantly improve the 24-hour survival rate of mice. Compared to RU21, it was more effective. The potential mechanisms were probably mediated by decreasing the β-EP and LENK levels in the brain and increasing the concentrations of ADH, ALDH and P450 to prevent acute alcoholism in mice. Besides, its protective effects on alcoholic liver injury were likely mediated through increasing SOD activity, decreasing MDA activity and the release of inflammatory factors TNF-α and IL-8, and lowering serum ALT, AST and ALP activities.
